# Caries-related risk factors of obesity among 18-year-old adolescents in Hong Kong: a cross-sectional study nested in a cohort study

**DOI:** 10.1186/s12903-018-0657-5

**Published:** 2018-11-20

**Authors:** Ling-Wei Li, Hai Ming Wong, Akanksha Gandhi, Colman Patrick McGrath

**Affiliations:** 1 Paediatric Dentistry & Orthodontics, Faculty of Dentistry, The University of Hong Kong, 2/F Prince Philip Dental Hospital, 34 Hospital Road, Hong Kong SAR, China; 2 Food and Nutritional Science, School of Biological Sciences, The University of Hong Kong, Hong Kong SAR, China; 3 Periodontology & Public health, Faculty of Dentistry, The University of Hong Kong, Hong Kong SAR, China

**Keywords:** Risk factors, Obesity, Caries, Adolescents

## Abstract

**Background:**

Socio-economic status, health awareness, and dietary habits have been reported as common risk factors of dental caries and obesity. The present study aimed to explore shared mediators between caries and obesity and to estimate the effects of caries-related factors on adiposity.

**Methods:**

This was a cross-sectional study among adolescents aged 18 years. The study was nested in a population-representative cohort of Chinese in Hong Kong. The number of decayed, missing, and filled permanent teeth (DMFT) was recorded during the oral examinations. Body Mass Index (BMI), Waist Circumference (WC), Waist-Hip-Ratio (WHR), Waist-Height-Ratio (WHtR), and Triceps Skinfold Thickness (TRSKF) were used as adiposity indices. Data on socio-economic status, oral health behavior (tooth brushing habit, use of fluoride toothpaste, dental flossing habit, and mouth rinse habit), and dietary record (frequency and amount of different food) were collected through self-completed questionnaires. Chi-square tests and binary logistic regressions were used for analysis.

**Results:**

Three hundred eighty-three participants were included. The mean (standard deviation, SD) of BMI, WC, WHR, WHtR, TRSKF, and DMFT were 21.26 (3.72), 69.11 (9.25), 0.77 (0.06), 0.42 (0.05), 15.72 (6.33), and 2.06 (2.43), respectively. Males were more likely to be overweight/obese than females. Various factors including gender, parental employment status, mouth rinse habit, frequency and amount of meat intake, frequency of oil intake, use of fluoride toothpaste, and DMFT were found significant (*p* < 0.05) in different final models of adiposity status.

**Conclusions:**

More mediators should be included in future research to elucidate mechanism of the association between caries and obesity.

## Background

Obesity among children and adolescents is one of the global public health concerns. It is well recognized that genetic factors, limited physical activities, and excess calories intake lead to overweight and obesity [1, 2]. In recent years, there is an increasing interest in the relationship between obesity and dental caries. Some researchers suggested a positive relationship [3] whereas inverse relationship was observed by others [4]. In this regard, there is the need to further explore the association between obesity and dental caries. In addition, an exploration of risk factors contributing to both diseases facilitates the making of a clear and sustainable global strategy for improving general and dental health. A number of potential genetic and environmental risk factors have been proposed, which included adiposity cell-factors, inflammatory factors, socio-economic status, health awareness, and diets [5, 6].

Dietary habit has been suggested as a possible risk factor for both caries and obesity [7]. There is a wealth of evidence suggesting dietary sugar as the most important causative factor for caries development. A well-known study in 1953 suggested that participants who consumed sugars more than four times per day had significantly increased numbers of decayed, missing, and filled permanent teeth (DMFT). The amount of sugar intake, on the other hand, was not associated with caries status [8]. However, other researchers found that the amount of sugar intake had a role as important as frequency of sugar intake in the progress of caries. In addition to leading to caries, sugar has also been recognized as one fundamental cause of overweight and obesity [9, 10]. World Health Organization (WHO) suggested that reducing energy-dense food that contained high amount of total fats and sugars and increasing intake of fruit, vegetables, legumes, whole grains, and nuts are important strategies for preventing overweight and obesity at individual level [2]. It is reasonable to assume that adolescents who consume sugar frequently or in abundance are predisposed to both adiposity and dental caries. Except for sugar, the effect of other diets, such as grain, fruit, and milk, on dental caries is much less investigated. It was revealed that high starch/low sugar diets reduced the risk of developing caries [11].

Health awareness is another possible risk factor for both caries and obesity. Nihtila et al. found that overweight populations had poorer oral health habit. For example, obese young adults brushed their teeth only once a day and visited dentists only for emergency treatment [12]. Franchini and his co-workers also agreed that the obese population had worse oral health awareness which resulted in poorer oral hygiene status and higher prevalence of gingivitis among children and adolescents aged 10 to 17 years [13].

Socio-economic status is an important consideration in most epidemiological studies. A systemic review showed that students from affluent families or regions were at an increased risk for obesity in sub-Saharan Africa [14]. On the contrary, a recent meta-analysis concluded that socio-economic status was inversely associated with prevalence of childhood overweight in developed areas [15]. Inconsistent conclusions have also been drawn regarding the association between dental caries and socio-economic status [3, 6, 16–19].

It was reported in Global Burden of Disease Study 2010 that 2.43 billion people still had untreated caries in their permanent teeth which were the most severe oral problem [20]. Economic burdens of oral diseases are substantial in developed areas and all over the world. Direct treatment costs were evaluated at USD$298 billion annually; the amount of direct and indirect costs of oral diseases were estimated at USD$442 billion in 2010 worldwide. Prevention of caries is not only physically but also economically beneficial to people [21]. Socio-economic status, oral health behavior, and diet are three possible environmental mediators accounting for the association between obesity and caries among children and adolescents. The present study aimed to explore shared mediators between caries and obesity and to estimate the effects of caries-related factors on adiposity.

## Methods

### Study design and study population

This was a cross-sectional study conducted in 2015. The study was nested in Hong Kong ‘Children of 1997’ birth cohort. All participants were born between April 1St 1997 and May 31th 1997. Forty-five out of 380 secondary schools were randomly selected. All 12-year-old students from these schools were invited to attend this surveys in 2010. This cohort was followed-up in their 15-year-old (2013) and 18-year-old (2015). A nutrition expert was invited for the questionnaire design, case measurement consistency and statistical analysis.

### Ethics, consent and permissions

This study was conducted according to the guidelines laid down in the Declaration of Helsinki and all procedures involving human subjects/patients were approved by the Institutional Review Board of the University of Hong Kong/Hospital Authority Hong Kong West Cluster (UW 15–178). A written consent from 18-year-old students, and parents/primary caregivers of 12- and 15-year-old students, and a verbal consent from 12- and 15-year-old students were obtained from all participants.

### Measurements

#### Oral health assessments

Participants’ caries and periodontal status were carefully examined at dental clinic settings following WHO guideline 2013 [22]. Intra-oral mirrors with LED lights and WHO CPI probes were provided to two trained and calibrated dentists for clinical examinations. No X-ray was taken to examine proximal caries. DMFT was used to record participants’ caries status. There were 10% (*n* = 43) participants randomly selected for re-examination to determine intra- and inter-examiner reliabilities.

#### Anthropometry assessments

Height, weight, WC, hip circumference, and TRSKF of the participants were evaluated as anthropometric assessments. All participants were lightly dressed and wear no shoes. Body height was assessed with stadiometer and body weight was recorded with using a self-zeroing digital scale. TRSKF was measured in the midline of the posterior aspect of the arm, over the triceps muscle. HC was carried out at the level of maximum extension of the buttocks in a horizontal plane. WC was taken at the level of the narrowest part of the torso. Intra- and inter-examiner reliability was evaluated by 10% random selected repeated assessment.

#### Self-administered questionnaires

The self-completed questionnaires were issued to participants after they received oral health examinations. Information on socio-economic status, dietary habits, and oral health behavior were collected. An expert nutritionist (AG) was consulted for the questionnaire design and diagnostic reliability of dietary part. An interviewer who had been trained by the expert to collect dietary data was ready to assist the participants whenever necessary. The participants were asked about the frequency and amount of intake of grains, vegetables, fruits, milk, meat, oil, and sweet as the expert suggested. Based on findings from several meta-analyses [23, 24], these types of food were included into the “Food pyramid” by the WHO, Food and Agriculture Organization (FAO), and the United States Department of Agriculture to prevent obesity and dental caries. The criteria of each serving followed the guideline of Hong Kong government [25]. Photos of food in each serving were shown to the participants if they were unable to determine the amount of food intake. Other information such as family income, parental employment status, frequency of tooth brushing, frequency of dental flossing, and frequency of mouth rinse were obtained in the self-completed questionnaires. Participants whose parents were both full-time employed were defined as “full-time employment group”, while other participants were defined as “non-full-time employment group”. Household monthly income were divided into “low-income group” (family income ≤ HK$10,000), “medium-income group” (HK$10,000 < family income ≤ HK$30,000), and “high-income group” (family income > HK$30,000).

### Statistical analysis

According to previous studies, it was reported that the odds ratio was 1.40 for having a dental caries experience with an increase of 1 unit in BMI *z*-score [26], and the prevalence of dental caries was at about 38% among 12-year-old students in Hong Kong [25]. The statistical power is set at 80% and the level of significance was set at 0.05. The sample size of 480 was needed in the baseline study. The Kolmogorov-Smirnov tests revealed that the values of BMI, WC, WHR, WHtR, TRSKF, and DMFT were not normally distributed (*P* < 0.05). Based on adiposity indices, participants were divided into two groups. Participants were also divided into the caries-free group (DMFT = 0) and caries group (DMFT > 0) according to values of DMFT.

Bivariate relationships between obesity indices and different independent variables were explored through Chi-square tests. The choice of dietary variables which was used in the regression models and the threshold points were reviewed by the nutritionist expert. Differences between the caries and caries-free group with respect to these variables were also evaluated by Chi-square tests. Binary logistic regressions with backward elimination were employed to explore the effect of risk factors on various obesity indices. All independent variables were included into the statistical models, except for the frequency and amount of food intake, which were put into different models to remove the effect of multicollinearity. Intra- and inter-examiner reliability in assessment of obesity and DMFT were determined through intraclass correlation coefficient (ICC).

## Results

In the baseline survey, 668 participants were enrolled in 2010. Of them, 436 (65.3%) participated in the first follow-up in 2013 at age 15. There were 383 18-year-old adolescents (56.1% girls and 43.9% boys) included in this third-round study in 2015. Two boys did not complete the questionnaires and were excluded from analysis. Finally, data from 215 females and 166 males were used for analysis.

The mean (standard deviation, SD) BMI, WC, WHR, WHtR, TRSKF, and DMFT were 21.26 (3.72), 69.11 (9.25), 0.77 (0.06), 0.42 (0.05), 15.72 (6.33), and 2.06 (2.43), respectively. Among the five adiposity indices, BMI, WC, and WHR of boys were significantly higher than those of girls (Mann-Whitney U tests, *P* < 0.05). TRSKF and DMFT of girls were significantly smaller than those of boys (Mann-Whitney U tests, *P* < 0.05) (Data not shown in the tables). The ICC values for DMFT, height, weight, WC, hip circumference, and TRSKF ranged between 0.94 and1.00, representing excellent agreement.

There were 79.6% of participants in the “full-time employment group” who were underweight/normal weight. The percentage was significantly (*P* = 0.009) lower than that among the “non-full-time employment group” (91.8%). The probability of being in the lower TRSKF group was significantly (*P* = 0.046) higher for participants in the “non-full-time employment group” (56.1%) than participants in the other group (43.3%). No significant differences in adiposity status were observed among participants with different levels of household monthly income (Table 1). With regard to oral health behavior, there was a significant difference in distribution of levels of BMI between adolescents who brushed their teeth less than once a day and those who brushed their teeth at least once a day. Students who brushed teeth less frequently had a higher probability of being overweight/obese (21.9%) compared to those who brushed more frequently (12.7%) (*P* = 0.023). Similar patterns were observed for WC (57.0% vs 47.2%), WHR (57.0% vs 46.4%), and WHtR (56.1% vs 47.2%), although the *P*-values were not statistically significant (WC: *P* = 0.079, WHR: *P* = 0.059, and WHtR: *P* = 0.110). However, it was surprising to find that 45.3% of participants who used fluoride toothpaste had lower WHtR. This percentage was significantly (*P* = 0.034) lower than that of those who did not use fluoride toothpaste or did not know what fluoride toothpaste was (56.2%). For dental flossing habit or mouth rinse habit, there was no difference in adiposity status between participants in the better hygiene behavior group and in the worse hygiene behavior group or between participants in the caries and in the caries-free group (Table 1).

**Table 1:** Association of socio-demographic, oral health behavior, and caries status with adiposity status

Variable	n (%)	BMI		WC		WHR		WHtR		TRSKF	
		Underweight + normal weight n (%)	Overweight + Obese n (%)	≤median n (%)	>median n (%)	≤median n (%)	>median n (%)	≤median n (%)	>median n (%)	≤median n (%)	>median n (%)
Gender			*P* = 0.003**		*P* = 0.964		*P* = 0.943		*P* = 0.872		*P* = 0.822
Male	166 (43.7%)	130 (78.3%)	36 (21.7%)	83 (50.0%)	83 (50.0%)	84 (50.6%)	82 (49.4%)	84 (50.6%)	82 (49.4%)	83 (50.0%)	83 (50.0%)
Female	215 (56.3%)	192 (89.3%)	23 (10.7%)	107 (49.8%)	108 (50.2%)	108 (50.2%)	107 (49.8%)	107 (49.8%)	108 (50.2%)	110 (51.2%)	105 (48.8%)
Full time employment for both parents			*P* = 0.009**		*P* = 0.913		*P* = 0.835		*P* = 0.572		*P* = 0.046*
Yes	157 (61.6%)	125 (79.6%)	32 (20.4%)	79 (50.3%)	78 (49.7%)	79 (50.3%)	78 (49.7%)	81 (51.6%)	76 (48.4%)	68 (43.3%)	89 (56.7%)
No	98 (38.4%)	90 (91.8%)	8 (8.2%)	50 (51.0%)	48 (49.0%)	48 (49.0%)	50 (51.0%)	47 (48.0%)	51 (52.0%)	55 (56.1%)	43 (43.9%)
Household monthly income			*P* = 0.707		*P* = 0.896		*P* = 0.732		*P* = 0.732		*P* = 0.716
< HK$ 10,000	28 (11.2%)	23 (82.1%)	5 (17.9%)	13 (46.4%)	15 (53.6%)	12 (42.9%)	16 (57.1%)	12 (42.9%)	16 (57.1%)	15 (53.6%)	13 (46.4%)
HK$ 10,000-HK$30,000	143 (57.0%)	120 (83.9%)	23 (16.1%)	73 (51.0%)	70 (49.0%)	72 (50.3%)	71 (49.7%)	72 (50.3%)	71 (49.7%)	66 (46.2%)	77 (53.8%)
> HK$ 30,000	80 (31.9%)	70 (87.5%)	10 (12.5%)	41 (51.3%)	39 (48.8%)	41 (51.3%)	39 (48.8%)	41 (51.3%)	39 (48.8%)	40 (50.0%)	40 (50.0%)
Tooth brushing habit			*P* = 0.023*		*P* = 0.079		*P* = 0.059		*P* = 0.110		*P* = 0.402
Less than once a day	114 (29.9%)	89 (78.1%)	25 (21.9%)	49 (43.0%)	65 (57.0%)	49 (43.0%)	65 (57.0%)	50 (43.9%)	64 (56.1%)	54 (47.4%)	60 (52.6%)
At least once a day	267 (70.1%)	233 (87.3%)	34 (12.7%)	141 (52.8%)	126 (47.2%)	143 (53.6%)	124 (46.4%)	141 (52.8%)	126 (47.2%)	139 (52.1%)	128 (47.9%)
Use of fluoride toothpaste			*P* = 0.366		*P* = 0.238		*P* = 0.429		*P* = 0.034*		*P* = 0.774
Yes	212 (55.6%)	176 (83.0%)	36 (17.0%)	100 (47.2%)	112 (52.8%)	103 (48.6%)	109 (51.4%)	96 (45.3%)	116 (54.7%)	106 (50.0%)	106 (50.0%)
No	169 (944.4%)	146 (86.4%)	23 (13.6%)	90 (53.3%)	79 (46.7%)	89 (52.7%)	80 (47.3%)	95 (56.2%)	74 (43.8%)	87 (51.5%)	82 (48.5%)
Dental flossing habit			*P* = 0.431		*P* = 0.862		*P* = 0.344		*P* = 0.969		*P* = 0.818
Less than once a day	140 (37.0%)	121 (86.4%)	19 (13.6%)	69 (49.3%)	71 (50.7%)	75 (53.6%)	65 (46.4%)	70 (50.0%)	70 (50.0%)	72 (51.4%)	68 (48.6%)
At least once a day	241 (63%)	201 (83.4%)	40 (16.6%)	121 (50.2%)	120 (49.8%)	117 (48.5%)	124 (51.5%)	121 (50.2%)	120 (49.8%)	121 (50.2%)	120 (49.8%)
Mouth Rinse			*P* = 0.131		*P* = 0.434		*P* = 0.336		*P* = 0.383		*P* = 0.693
Less than once a day	166 (43.6%)	135 (81.3%)	31 (18.7%)	79 (47.6%)	87 (52.4%)	79 (47.6%)	87 (52.4%)	79 (47.6%)	87 (52.4%)	86 (51.8%)	80 (48.2%)
At least once a day	215 (56.4%)	187 (87.0%)	28 (13.0%)	111 (51.6%)	104 (48.4%)	113 (52.6%)	102 (47.4%)	112 (52.1%)	103 (47.9%)	107 (49.8%)	108 (50.2%)
DMFT			*P* = 0.755		*P* = 0.232		*P* = 0.541		*P* = 0.186		*P* = 0.603
DMFT = 0	149 (39.1%)	127 (85.2%)	22 (14.8%)	80 (53.7%)	69 (46.3%)	78 (52.3%)	71 (47.7%)	81 (54.4%)	68 (45.6%)	120 (51.7%)	112 (48.3%)
DMFT > 0	232 (60.9%)	195 (84.1%)	37 (15.9%)	110 (47.4%)	122 (52.6%)	114 (49.1%)	118 (50.9%)	110 (47.4%)	122 (52.6%)	73 (49.0%)	76 (51.0%)

Abbreviations: *DMFT* the number of decayed, missing, and filled permanent teeth, *BMI* body mass index, *WC* waist circumference, *WHR* waist-hip ratio, *WHtR* waist-height ratio, *TRSKF* triceps skinfold thickness

BMI was classified into underweight/normal weight group vs overweight/obese group; WC, WHR, WHtR, and TRSKF were dichotomized by the median values

*P*: *P < 0.05, **P < 0.01

*P* values were calculated by Chi-square test

Most of the 18-year-old participants did not take their food following the guidelines of WHO and FAO. More than a quarter of participants (26.8%) had vegetables less than once a day. There were 65.6 and 84.3% of participants who consumed fruits and milk, respectively, less than once a day. Regarding frequency of food intake, frequency of meat intake was the only factor related to BMI and WHR. The proportion of overweight/obese participants was significantly higher (*P* = 0.041) among those who took meat at least once a day (17.7%) than among those who consumed meat less than once a day (9.1%). Similarly, participants with higher frequency of meat intake also had higher probability (52.8%, *P* = 0.040) of being in the higher WHR group compared to those who consumed meat less frequently (40.8%) (Table 2).

**Table 2:** The relationship between frequency of food intake and obesity status

Variable	n (%)	BMI		WC		WHR		WHtR		TRSKF	
		Underweight + normal weight n (%)	Overweight + Obese n (%)	≤median n (%)	>median n (%)	≤median n (%)	>median n (%)	≤median n (%)	>median n (%)	≤median n (%)	>median n (%)
Grains			*P* = 0.388		*P* = 1.000		*P* = 0.721		*P* = 0.721		*P* = 0.666
Less than once a day	45 (11.8%)	40 (88.9%)	5 (11.1%)	22 (50.0%)	22 (50.0%)	21 (47.7%)	23 (52.3%)	21 (47.7%)	23 (52.3%)	21 (47.7%)	23 (52.3%)
At least once a day	336 (88.2%)	282 (83.9%)	54 (16.1%)	168 (50.0%)	168 (50.0%)	170 (50.6%)	166 (49.4%)	170 (50.6%)	166 (49.4%)	172 (51.2%)	164 (48.8%)
Vegetables			*P* = 0.566		*P* = 0.728		*P* = 0.521		*P* = 0.521		*P* = 0.870
Less than once a day	102 (26.8%)	88 (86.3%)	14 (13.7%)	52 (51.5%)	49 (48.5%)	48 (47.5%)	53 (52.5%)	48 (47.5%)	53 (52.5%)	52 (51.5%)	49 (48.5%)
At least once a day	279 (73.2%)	234 (83.9%)	45 (16.1%)	138 (49.5%)	141 (50.5%)	143 (51.3%)	136 (48.7%)	143 (51.3%)	136 (48.7%)	141 (50.5%)	138 (49.5%)
Fruits			*P* = 0.327		*P* = 1.000		*P* = 0.565		*P* = 0.720		*P* = 0.995
Less than once a day	250 (65.6%)	208 (83.2%)	42 (16.8%)	125 (50.0%)	125 (50.0%)	123 (49.2%)	127 (50.8%)	124 (49.6%)	126 (50.4%)	127 (50.8%)	123 (49.2%)
At least once a day	131 (34.4%)	114 (87.0%)	17 (13.0%)	65 (50.0%)	65 (50.0%)	68 (52.3%)	62 (47.7%)	67 (51.5%)	63 (48.5%)	66 (50.8%)	64 (49.2%)
Milk			*P* = 0.616		*P* = 0.778		*P* = 0.745		*P* = 0.604		*P* = 0.678
Less than once a day	321 (84.3%)	270 (84.1%)	51 (15.9%)	159 (49.7%)	161 (50.3%)	162 (50.6%)	158 (49.4%)	159 (49.7%)	161 (50.3%)	164 (51.3%)	156 (48.8%)
At least once a day	60 (15.7%)	52 (86.7%)	8 (13.3%)	31 (51.7%)	29 (48.3%)	29 (48.3%)	31 (51.7%)	32 (53.3%)	28 (46.7%)	29 (48.3%)	31 (51.7%)
Meat			*P* = 0.041*		*P* = 0.241		*P* = 0.040*		*P* = 0.380		*P* = 0.774
Less than once a day	99 (26.0%)	90 (90.9%)	9 (9.1%)	54 (55.1%)	44 (44.9%)	58 (59.2%)	40 (40.8%)	53 (54.1%)	45 (45.9%)	51 (52.0%)	47 (48.0%)
At least once a day	282 (74.0%)	232 (82.3%)	50 (17.7%)	136 (48.2%)	146 (51.8%)	133 (47.2%)	149 (52.8%)	138 (48.9%)	144 (51.1%)	142 (50.4%)	140 (49.6%)
Oil			*P* = 0.508		*P* = 0.918		*P* = 0.688		*P* = 0.543		*P* = 0.655
Less than once a day	170 (44.6%)	146 (85.9%)	24 (14.1%)	84 (49.7%)	85 (50.3%)	83 (49.1%)	86 (50.9%)	82 (48.5%)	87 (51.5%)	88 (52.1%)	81 (47.9%)
At least once a day	211 (55.4%)	176 (83.4%)	35 (16.6%)	106 (50.2%)	105 (49.8%)	108 (51.2%)	103 (48.8%)	109 (51.7%)	102 (48.3%)	105 (49.8%)	106 (50.2%)
Sweet			*P* = 0.853		*P* = 0.819		*P* = 0.328		*P* = 0.770		*P* = 0.790
Less than once a day	275 (72.2%)	233 (84.7%)	42 (15.3%)	138 (50.4%)	136 (49.6%)	142 (51.8%)	132 (48.2%)	139 (50.7%)	135 (49.3%)	138 (50.4%)	136 (49.6%)
At least once a day	106 (27.8%)	89 (84.0%)	17 (16.0%)	52 (49.1%)	54 (50.4%)	49 (46.2%)	57 (53.8%)	52 (49.1%)	54 (50.9%)	55 (51.9%)	51 (48.1%)

Abbreviations: *BMI* body mass index, *WC* waist circumference, *WHR* waist-hip ratio, *WHtR* waist-height ratio, *TRSKF* triceps skinfold thickness

BMI was classified into underweight/normal weight group vs overweight/obese group; WC, WHR, WHtR, and TRSKF were dichotomized by the median values

*P*: *P < 0.05

*P* values were calculated by Chi-square test

Relationship between the amount of food intake and various obesity indices was described in Table 3. Boys consumed significantly (*P <* 0.05) more grains, milk, and meat in each meal than girls. In contrast, the amount of sweet taken the by females was significantly (*P* = 0.001) higher than that taken by males. Participants with higher BMI, WC, and WHtR consumed significantly more (*P* < 0.05) meat than the less obese individuals. In addition, those who had higher WC consumed more vegetables (*P* = 0.02) than those with lower WC (Table 3).

**Table 3:** The relationship between the amount of food intake in each meal and obesity indices

Variable	Gender		BMI		WC		WHR		WHtR		TRSKF	
	Male	Female	Underweight + normal weight n (%)	Overweight + Obese n (%)	≤median n (%)	>median n (%)	≤median n (%)	>median n (%)	≤median n (%)	>median n (%)	≤median n (%)	>median n (%)
Grains		*P* < 0.001***		*P* = 0.791		*P* = 0.684		*P* = 0.701		*P* = 0.205		*P* = 0.329
n Mean (SD) Median	165 1.72 (0.71) 2.00	212 1.29 (0.62) 1.00	319 1.49 (0.70) 1.00	58 1.45 (0.65) 1.00	189 1.50 (0.71) 1.00	188 1.47 (0.68) 1.00	189 1.49 (0.67) 1.00	188 1.48 (0.72) 1.00	189 1.52 (0.71) 1.00	188 1.44 (0.68) 1.00	191 1.52 (0.70) 1.00	186 1.45 (0.68) 1.00
Vegetables		*P* = 0.478		*P* = 0.238		*P* = 0.020**		*P* = 0.582		*P* = 0.097		*P* = 0.896
n Mean (SD) Median	163 1.48 (0.75) 1.00	213 1.52 (0.83) 1.00	320 1.49 (0.80) 1.00	56 1.60 (0.72) 1.50	190 1.47 (0.93) 1.00	186 1.55 (0.62) 1.50	189 1.53 (0.95) 1.00	187 1.49 (0.60) 1.00	190 1.49 (0.94) 1.00	186 1.53 (0.62) 1.50	192 1.50 (0.71) 1.00	184 1.52 (0.88) 1.00
Fruits		*P* = 0.095		*P* = 0.644		*P* = 0.332		*P* = 0.743		*P* = 0.350		*P* = 0.661
n Mean (SD) Media n	166 1.06 (0.43) 1.00	211 1.13 (0.42) 1.00	318 1.097 (0.42) 1.00	59 1.14 (0.48) 1.00	188 1.08 (0.42) 1.00	189 1.12 (0.44) 1.00	189 1.10 (0.42) 1.00	188 1.11 (0.44) 1.00	188 1.09 (0.44) 1.00	189 1.12 (0.42) 1.00	191 1.09 (0.41) 1.00	186 1.12 (0.45) 1.00
Milk		*P* = 0.015**		*P* = 0.655		*P* = 0.159		*P* = 0.435		*P* = 0.314		*P* = 0.729
n Mean (SD) Median	163 1.14 (0.47) 1.00	213 1.05 (0.40) 1.00	319 1.09 (0.43) 1.00	57 1.08 (0.46) 1.00	188 1.06 (0.36) 1.00	188 1.122 (0.50) 1.00	189 1.06 (0.37) 1.00	187 1.12 (0.49) 1.00	189 1.06 (0.36) 1.00	187 1.12 (0.50) 1.00	192 1.07 (0.41) 1.00	184 1.11 (0.46) 1.00
Meat		*P* < 0.001***		*P* = 0.036*		*P* = 0.001**		*P* = 0.018		*P* = 0.013*		*P* = 0.204
n Mean (SD) Median	164 3.23 (1.78) 3.00	213 2.47 (1.70) 2.00	320 2.73 (1.75) 2.00	57 3.19 (1.87) 3.00	189 2.54 (1.46) 2.00	188 3.06 (2.01) 3.00	190 2.62 (1.50) 2.00	187 2.98 (2.00) 3.00	190 2.59 (1.46) 2.00	187 3.01 (2.02) 3.00	192 2.79 (2.00) 1.00	185 2.81 (1.51) 2.50
Oil		*P* = 0.878		*P* = 0.214		*P* = 0.313		*P* = 0.639		*P* = 0.280		*P* = 0.382
n Mean (SD) Median	161 1.37 (0.74) 1.00	206 1.38 (0.69) 1.00	311 1.36 (0.72) 1.00	56 1.46 (0.67) 1.00	184 1.32 (0.59) 1.00	183 1.43 (0.82) 1.00	187 1.37 (0.60) 1.00	180 1.38 (0.81) 1.00	184 1.32 (0.58) 1.00	183 1.43 (0.82) 1.00	187 1.32 (0.60) 1.00	180 1.43 (0.81) 1.00
Sweet		*P* = 0.001**		*P* = 0.236		*P* = 0.819		*P* = 0.939		*P* = 0.360		*P* = 0.595
n Mean (SD) Median	165 2.90 (2.53) 2.00	209 3.82 (3.48) 3.00	316 3.52 (3.26) 2.00	58 2.85 (2.18) 2.00	187 3.37 (2.98) 2.00	187 3.46 (3.27) 2.00	190 3.40 (2.98) 2.00	184 3.44 (3.28) 2.00	188 3.29 (2.90) 2.00	186 3.54 (3.34) 2.00	188 3.36 (3.01) 2.00	186 3.47 (3.24) 2.00

Abbreviations: *BMI* body mass index, *WC* waist circumference, *WHR* waist-hip ratio, *WHtR* waist-height ratio, *TRSKF* triceps skinfold thickness

BMI was classified into underweight/normal weight group vs overweight/obese group; WC, WHR, WHtR, and TRSKF were dichotomized by median values

*P < 0.05, **P < 0.01, ***P < 0.001

*P*: Non-parametric tests, Mann-Whitney U test/Kruskal-Wallis test

Oral examination of caries status revealed that 60.9% of participants had caries (data not shown). The proportion of participants with caries was significantly higher (*P* = 0.033) among females (65.6%) than among males (54.8%). Participants who consumed sweet at least once a day had significantly (*P* = 0.027) higher prevalence of caries (69.8%) than those who consumed sweet less than once a day (57.5%). There was no significant difference (*P* > 0.05) in caries status among participants with different socio-demographic status, oral health behaviors, frequency of food intake except for sweet, or amount of food intake (Table 4).

**Table 4:** Association of socio-demographic, oral health behavior, and food intake record with dental caries experience

Variable	*n* (%)	*n* (%) (DMFT = 0)	*n* (%) (DMFT > 0)	*P*
Gender^a^				*P* = 0.033*
Male	166 (43.7%)	75 (45.2%)	91 (54.8%)	
Female	215 (56.3%)	74 (34.4%)	141 (65.6%)	
Full time employment for both parents^a^				*P* = 0.660
Yes	157 (61.6%)	62 (39.5%)	95 (60.5%)	
No	98 (38.4%)	36 (36.7%)	62 (63.3%)	
Household monthly income^a^				*P* = 0.592
< HK$ 10,000	28 (11.2%)	19 (67.9%)	9 (32.1%)	
HK$ 10,000-HK$ 30,000	143 (57.0%)	89 (62.2%)	54 (37.8%)	
> HK$ 30,000	80 (31.9%)	46 (57.5%)	34 (42.5%)	
Tooth brushing habit^a^				*P* = 0.554
less than once a day	114 (29.9%)	72 (63.2%)	42 (36.8%)	
At least once a day	267 (70.1%)	180 (59.9%)	107 (40.1%)	
Use of fluoride toothpaste^a^				*P* = 0.848
Yes	212 (55.6%)	130 (61.3%)	82 (38.7%)	
No	169,944.4%)	102 (60.4%)	67 (39.6%)	
Dental flossing habit^a^				*P* = 0.479
Less than once a day	140 (37.0%)	82 (58.6%)	58 (41.4%)	
At least once a day	241 (63%)	150 (62.2%)	91 (37.8%)	
Mouth Rinse^a^				*P* = 0.388
Less than once a day	166 (43.6%)	97 (58.4%)	69 (41.6%)	
At least once a day	215 (56.4%)	135 (62.8%)	80 (37.2%)	
Frequency of Grains intake^a^				*P* = 0.648
Less than once a day	45 (11.8%)	19 (42.2%)	26 (57.8%)	
At least once a day	336 (88.2%)	130 (38.7%)	206 (61.3%)	
Frequency of vegetables intake^a^				*P* = 0.792
Less than once a day	102 (26.8%)	41 (40.2%)	61 (59.8%)	
At least once a day	279 (73.2%)	108 (38.7%)	171 (61.3%)	
Frequency of fruits intake^a^				*P* = 0.405
Less than once a day	250 (65.6%)	94 (37.6%)	156 (62.4%)	
At least once a day	131 (34.4%)	55 (42.0%)	76 (58.0%)	
Frequency of milk intake^a^				*P* = 0.191
Less than once a day	321 (84.3%)	121 (37.7%)	200 (62.3%)	
At least once a day	60 (15.7%)	28 (46.7%)	32 (53.3%)	
Frequency of meat intake^a^				*P* = 0.759
Less than once a day	99 (26.0%)	40 (40.4%)	59 (59.6%)	
At least once a day	282 (74.0%)	109 (38.7%)	173 (61.3%)	
Frequency of oil intake^a^				*P* = 0.247
Less than once a day	170 (44.6%)	61 (35.9%)	109 (64.1%)	
At least once a day	211 (55.4%)	88 (41.7%)	123 (58.3%)	
Frequency of sweet intake^a^				*P* = 0.027*
Less than once a day	275 (72.2%)	117 (42.5%)	158 (57.5%)	
At least once a day	106 (27.8%)	32 (30.2%)	74 (69.8%)	
	Mean(SD)	Mean(SD)	Mean(SD)	
Amount of Grains intake^b^	1.48 (0.69)	1.51 (0.75)	1.46 (0.66)	*P* = 0.788
Amount of vegetables intake^b^	1.51 (0.80)	1.49 (0.66)	1.52 (0.87)	*P* = 0.951
Amount of fruits intake^b^	1.10 (0.43)	1.10 (0.43)	1.11 (0.43)	*P* = 0.618
Amount of milk intake^b^	1.09 (0.43)	1.10 (0.42)	1.08 (0.44)	*P* = 0.707
Amount of meat intake^b^	2.80 (1.77)	2.82 (1.99)	2.78 (1.62)	*P* = 0.762
Amount of oil intake^b^	1.38 (0.71)	1.38 (0.72)	1.37 (0.71)	*P* = 0.837
Amount of sweet intake^b^	3.42 (3.13)	3.23 (2.80)	3.53 (3.32)	*P* = 0.201

Abbreviations: DMFT, the number of decayed, missing, and filled permanent teeth

*P < 0.05

^a^*P* values were calculated through Chi-square test; ^b^*P* values were calculated through Mann-Whitney Test

Significant association was found between various independent variables and obesity indices (Table 5). Males were 2.72 times as likely (95% CI, 1.23–6.05; *P* = 0.014) to be overweight/obese as females do. Participants in the “full-time employment group” were 4.03 times as likely (95% CI, 1.58–10.26; *P* = 0.003) to be overweight/obese as those in the “non-full-time employment group”. Participants who rinsed their mouths less than once a day had higher chance (Odds Ratio (OR) = 2.31; 95% CI, 1.08–4.96; *P* = 0.032) of being overweight/obese than those who rinsed their mouths more frequently (*P* = 0.032). Those who had meat (OR = 0.32; 95% CI, 0.10–0.99; *P* = 0.048) or oil (OR = 0.37; 95% CI, 0.16–0.87; *P* = 0.022) less than once a day were less likely to have BMI ≥ 25 kg/m^2^ than the more frequent consumers. Higher DMFT was associated with increased chance of being overweight/obese (OR = 1.18; 95% CI, 1.02–1.37; *P* = 0.031). As the only significant independent variable identified, DMFT was found positively associated with WHR (OR = 1.11; 95% CI, 1.00–1.23; *P* = 0.044). Regarding TRSKF, participants in the “full-time employment group” had significant higher chance of being in the higher TRSKF group than those in the “non-full-time employment group” (OR = 1.78; 95% CI, 1.06–2.99; *P* = 0.028). There were no independent variables significantly associated with WC or WHtR.

**Table 5:** The relationship between adiposity status and the significant independent variables in Model 1

Independent Variables	BMI			WHR			TRSKF	
	*OR*	*95% CI*	*P*	*OR*	*95% CI*	*P*	*OR*	*95% CI*
Gender								
Male	2.72	1.23, 6.05	0.014*					
Female	1.00							
Full time employment for both parents								
Yes	4.03	1.58, 10.26	0.003**				1.78	1.06, 2.99
No	1.00						1.00	
Mouth rinse								
Less than once a day	2.31	1.08, 4.96	0.032*					
More than once a day	1.00							
Frequency of meat intake								
Less than once a day	0.32	0.10, 0.99	0.048*					
More than once a day	1.00							
Frequency of oil intake								
Less than once a day	0.37	0.16, 0.87	0.022*					
More than once a day	1.00							
DMFT	1.18	1.02, 1.37	0.031*	1.11	1.00, 1.23	0.044*		

Abbreviation: *DMFT* decayed, missing, and filled permanent teeth, *CI* conference interval, *BMI* body mass index, *WHR* waist-hip ratio, *TRSKF* triceps skinfold thickness

Dependent variable (categorical data): Adiposity indices; BMI was classified into underweight/normal weight group vs overweight/obese group (event group); WHR and TRSKF were classified by median values (higher 50% is event group)

Independent variable: DMFT (continuous data), gender, full time employment of both parents, household monthly income, frequency of tooth brushing, use of fluoride toothpaste, frequency of mouth rinse, frequency of flossing, frequency of grains intake, frequency of vegetables intake, frequency of fruits intake, frequency of milk intake, frequency of meat intake, frequency of oil intake, and frequency of sweet intake (categorical data)

In the final models of WC and WHtR, all independent variables were removed in the final model with α set at 0.05. The data of WC and WHtR were not presented here

*P < 0.05, **P < 0.01. *P* values were calculated through binary logistic regression

When the amount of food intake was included and analyzed in other models, different set of variables were found significant in the final models (Table 6). Regarding BMI, males were 3.13 times as likely to be overweight/obese as females (95% CI, 1.39–7.03; *P* = 0.006). Participants whose both parents were full-time employed were more likely to be overweight/obese than those in the “non-full-time employment group” (OR = 3.43; 95% CI, 1.32–8.91; *P* = 0.011). Participants with higher DMFT had significantly increased probability of having BMI ≥ 25 kg/m^2^ (OR = 1.18; 95% CI, 1.02–1.36; *P* = 0.026). Regarding WC, the amount of meat intake was the only variable significant in the final model. Consuming more meat was associated with significantly increased probability to have higher WC (OR = 1.30; 95% CI, 1.07–1.60; *P* = 0.010). Regarding WHR, each unit increase in DMFT was associated with 12% increase in the chance of having higher WHR (OR = 1.12; 95% CI, 1.01–1.25; *P* = 0.027). Regarding WHtR, the chance for those who used fluoride toothpaste to have higher WHtR was significantly higher than those who did not use fluoride toothpaste or did not know what fluoride toothpaste was (OR = 1.71; 95% CI, 1.00–2.92; *P* = 0.049). Consuming higher amount of meat was significantly associated with increased probability of having higher WHtR (OR = 1.25, 95% CI, 1.02–1.52; *P* = 0.029). Regarding TRSKF, those in the “full-time employment group” had significant higher chance to have higher TRSKF compared to those in the “non-full-time employment group” (OR = 1.83; 95% CI, 1.07–3.13; *P* = 0.027).

**Table 6:** The relationship between adiposity status and the significant independent variables in Model 2

Independent Variables	BMI			WC			WHR			WHtR	TRSKF			
	*OR*	*95% CI*	*P*	*OR*	*95% CI*	*P*	*OR*	*95% CI*	*P*	*OR*	*95% CI*	*P*	*OR*	*95% CI*
Gender														
Male	3.13	1.39, 7.03	0.006**											
Female	1.00													
Full time employment for both parents														
Yes	3.43	1.32, 8.91	0.011*										1.83	1.07, 3.13
No	1.00												1.00	
Use of fluoride toothpaste														
Yes										1.71	1.00, 2.92	0.049*		
No										1.00				
Amount of meat				1.30	1.07, 1.60	0.010*				1.25	1.02, 1.52	0.029*		
DMFT	1.18	1.02, 1.36	0.026*				1.12	1.01, 1.25	0.027*					

Abbreviation: *DMFT* decayed, missing, and filled permanent teeth, *CI* conference interval, *BMI* body mass index, *WHR* waist-hip ratio, *WHtR* Waist-height ratio, *TRSKF* triceps skinfold thickness

Dependent variable (categorical data): Adiposity indicess; BMI was classified into underweight/normal weight group vs overweight/obese group (event group); WHR and TRSKF was classified by median values (higher 50% is event group)

Independent variable: DMFT (continuous data), gender, full time employment of both parents, household monthly income, frequency of tooth brushing, use of fluoride toothpaste, frequency of mouth rinse, frequency of flossing, amount of grains intake, amount of vegetables intake, amount of fruit intake, amount of milk intake, amount of meat intake, amount of oil intake, and amount of sweet intake (categorical data)

*P < 0.05, **P < 0.01

*P* values were calculated through binary logistic regression

## Discussion

Only 15.5% of participants were overweight/obese based on International Obesity Task Force (IOTF) cut-offs (25.00 kg/m^2^ ≥ BMI > 30.00 kg/m^2^ was defined as overweight and BMI ≥ 30.00 kg/m^2^ was defined as obesity in 18-year-old adolescents) [27], whereas the percentage raised to 25.5% according to the WHO classification of BMI in adult Asians which defined overweight as BMI ≥ 23.00 kg/m^2^ [28]. The cut-off points played an important role in the incidence rate of obesity. It was assumed that socio-economic status, health awareness, and dietary habit were important common contributors for obesity and caries. However, our findings suggested gender as the only common factor underlying the two diseases (Table 1-4). Sugar was supposed to play a key role in the development of both caries and obesity, hence it was not surprising to find that frequency of sweet intake was associated with caries experience. Unexpectedly, frequency and amount of sweet intake were found not related to adiposity status. This might be explained by the frequent exercise among adolescents, leading to energy balance in spite of the larger amount of sweet intake. A recent systemic review suggested inconsistent associations between sugar-sweetened beverage intake and obesity risk after adjusting for energy balance among different age groups [29]. For the present analysis, girls consumed significantly more sweets but fewer grains, milk, and meat than boys. Additionally, it seemed that young girls might have biased estimation of their body images, which led to eating disturbances [30]. Furthermore, it was also shown that sensory liking for sweet was related with a decreased risk of obesity [31].

Our study indicated that overweight or obese participants might have worse brushing habit. Nihtila et al. [12] and Franchini et al. [13] also agreed that obese individuals should improve their oral health awareness. This view seems to support the theory that health awareness mediates the association between dental caries and adiposity. However, it is unexpected that caries status was not associated with oral health behavior in our analysis. This was possible in that caries development is a complex process and there are factors other than oral hygiene status that may have an impact on the process. Hong Kong is a well-developed region with all residents having access to 0.05 ppm fluoridated water. Children from 6 to 12 years are also exposed to School Dental Care Service of Hong Kong Government which include oral health education, oral examination, preventive dental treatment, and basic restorative dental treatment [32]. Furthermore, more than 70% of adolescents in our survey had sweet less than once a day. These factors may have played critical roles in reducing the occurrence of caries among Hong Kong adolescents.

From the binary logistic regressions, it was found that gender, parental employment status, mouth rinse habit, frequency and amount of meat intake, frequency of oil intake, use of fluoride toothpaste, and DMFT were related with adiposity. Caries experience was directly associated with BMI and WHR. It should be noticed that DMFT was the only variable significantly associated with WHR. More efforts should be made to identify and confirm such association. Generally speaking, two recommendations are made to obtain a fuller understanding of this association. First, more confounders should be included in future analyses to help clarify unidentified risk factors underlying the association between caries and obesity. Second, laboratory studies are warranted to provide in-depth understanding of the association between caries and obesity.

Several limitations should be brought to attention. First of all, the commonly used three-day food frequency questionnaire cannot be used in this study due to time limitations. As a compromise, only selected questions were asked as suggested by the nutrition expert. A more detailed food frequency questionnaire should be employed in future studies to explore the relationship between obesity and caries. Secondly, there might be recall bias when participants were invited to estimate their dietary habit. Thirdly, soft drink consumption was not investigated in this study. A recent systemic review suggested inconsistent associations between sugar-sweetened beverage intake and obesity risk after adjusting for energy balance among different age groups [29]. Variables such as soft drink consumption and physical activity should be included in future studies.

## Conclusions

Various factors including socio-economic status, oral health habits, diet and caries experience were found to be associated with adiposity status in this cohort. As obese adolescents might lack health awareness and knowledge, related education and instructions are recommended to help this group of adolescents. Further exploration of the association between obesity and dental caries, and their common risk factors might inform optimal strategies for public health intervention programs to improve the health of adolescents.

## Abbreviations

BMI: Body Mass Index
DMFT: The number of decayed, missing, and filled permanent teeth
FAO: Food and Agriculture Organization
ICC: Intraclass Correlation Coefficient
IOTF: International Obesity Task Force
OR: Odds Ratio
SD: Standard Deviation
TRSKF: Triceps Skinfold Thickness
WC: Waist Circumference
WHO: World Health Organization
WHR: Waist-Hip-Ratio
WHtR: Waist-Height-Ratio
